# Supplementary material for: Optimization of Encapsulation by Ionic Gelation Technique of Cryoconcentrated Solution: A Response Surface Methodology and Evaluation of Physicochemical Characteristics Study
Source: Polymers (Basel). 2022 Mar 4;14(5):1031. doi: 10.3390/polym14051031 (PMC8914933; doi:10.3390/polym14051031)
Supplement: Supplementary file 1 [file polymers-14-01031-s001.zip › polymers-1607397-supplementary.pdf]

SUPPLEMENTARY MATERIALS

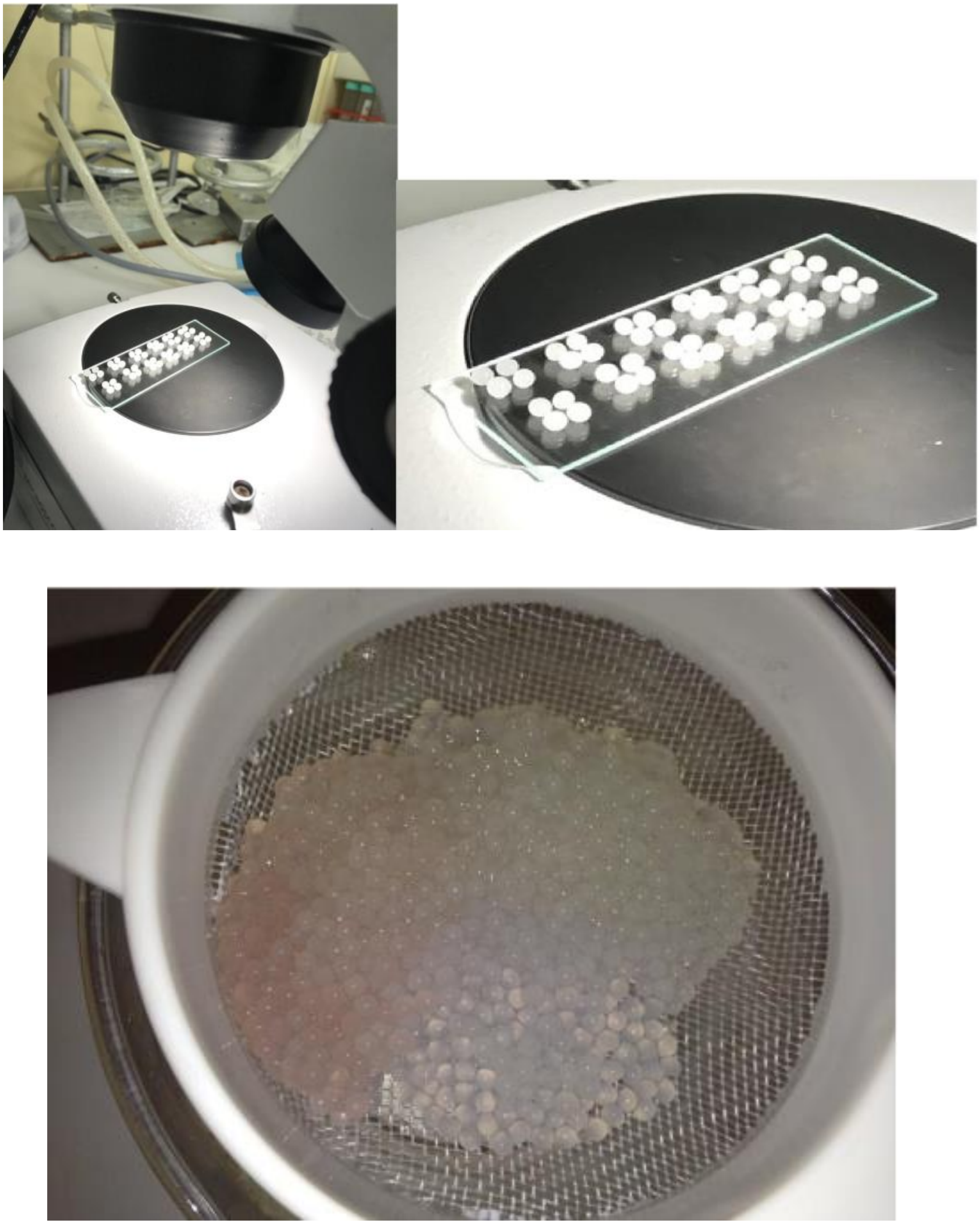

Figure S1. Photographs of the calcium alginate-cornstarch hydrogel beads filled with sucrose/acid gallic solution or cryoconcentrated solution.

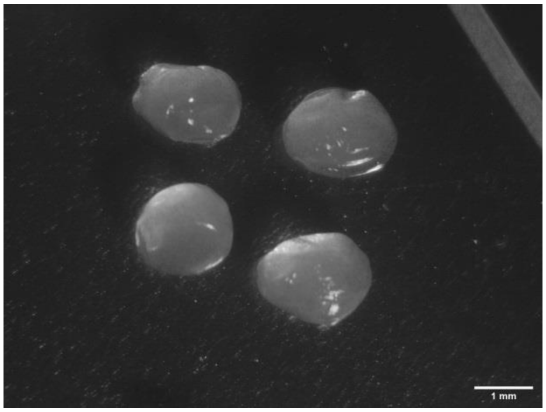

(**a**)

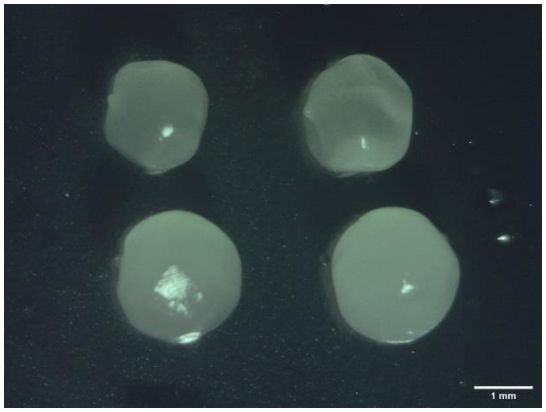

(**b**)

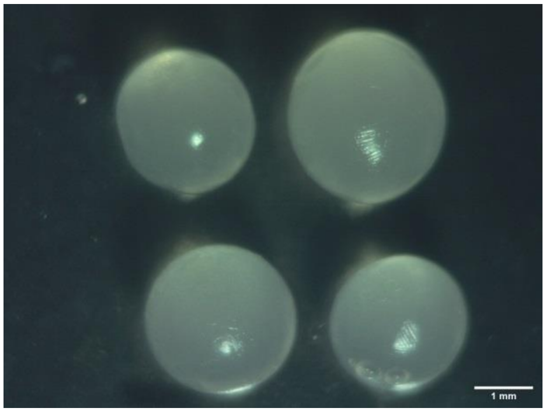

(**c**)

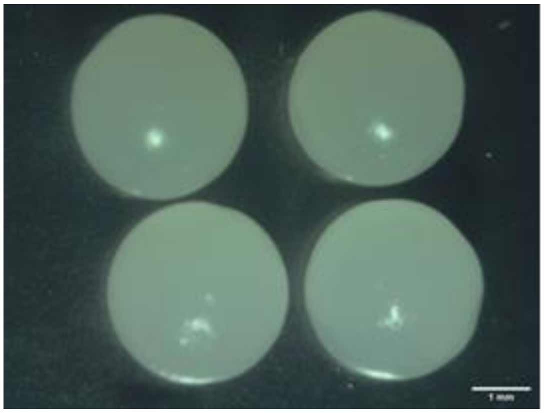

(**d**)

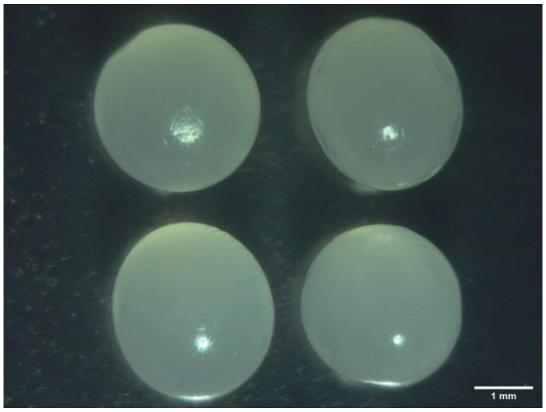

(**e**)

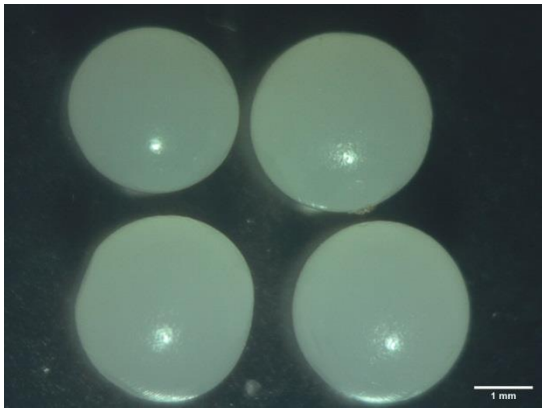

(**f**)

Figure S2. Microphotographs of calcium alginate-cornstarch hydrogel beads. Initial sucrose/GA solution with: (**a**) alginate at 1% (w/w) and cornstarch at 0.5% (w/w); (**b**) alginate at 1% (w/w) and cornstarch at 2% (w/w); (**c**) alginate at 2% (w/w) and cornstarch at 0.5% (w/w); (**d**) alginate at 2% (w/w) and cornstarch at 2% (w/w); (**e**) alginate at 2% (w/w) and cornstarch at 0.5% (w/w); (**f**) alginate at 2% (w/w) and cornstarch at 2% (w/w).

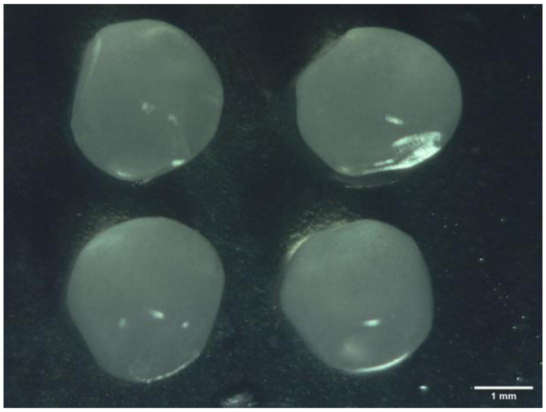

(**a**)

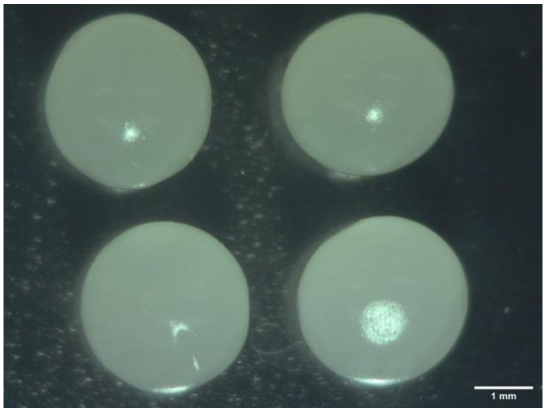

(**b**)

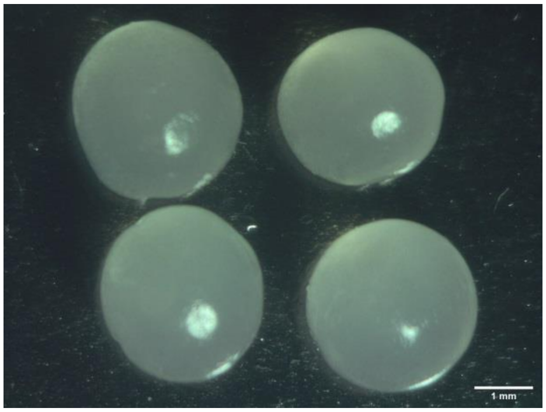

(**c**)

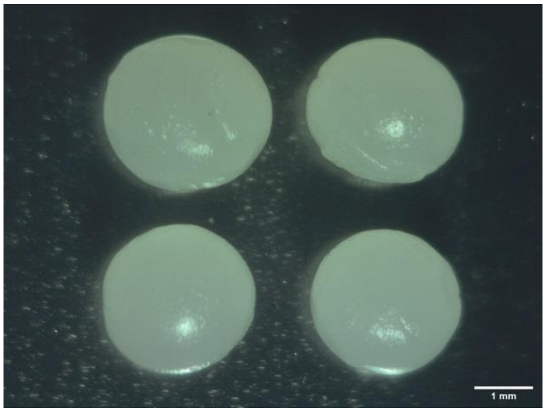

(**d**)

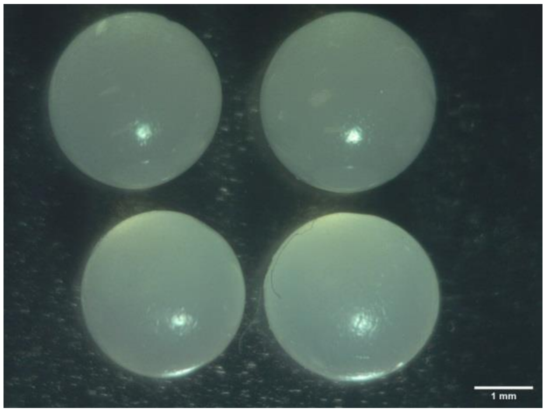

(**e**)

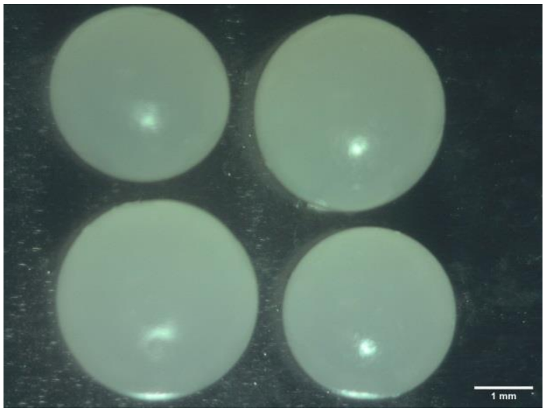

(**f**)

Figure S3. Microphotographs of calcium alginate-cornstarch hydrogel beads. Cryoconcentrated from cycle 1 with: (**a**) alginate at 1% (w/w) and cornstarch at 0.5% (w/w); (**b**) alginate at 1% (w/w) and cornstarch at 2% (w/w); (**c**) alginate at 2% (w/w) and cornstarch at 0.5% (w/w); (**d**) alginate at 2% (w/w) and cornstarch at 2% (w/w); (**e**) alginate at 2% (w/w) and cornstarch at 0.5% (w/w); (**f**) alginate at 2% (w/w) and cornstarch at 2% (w/w).

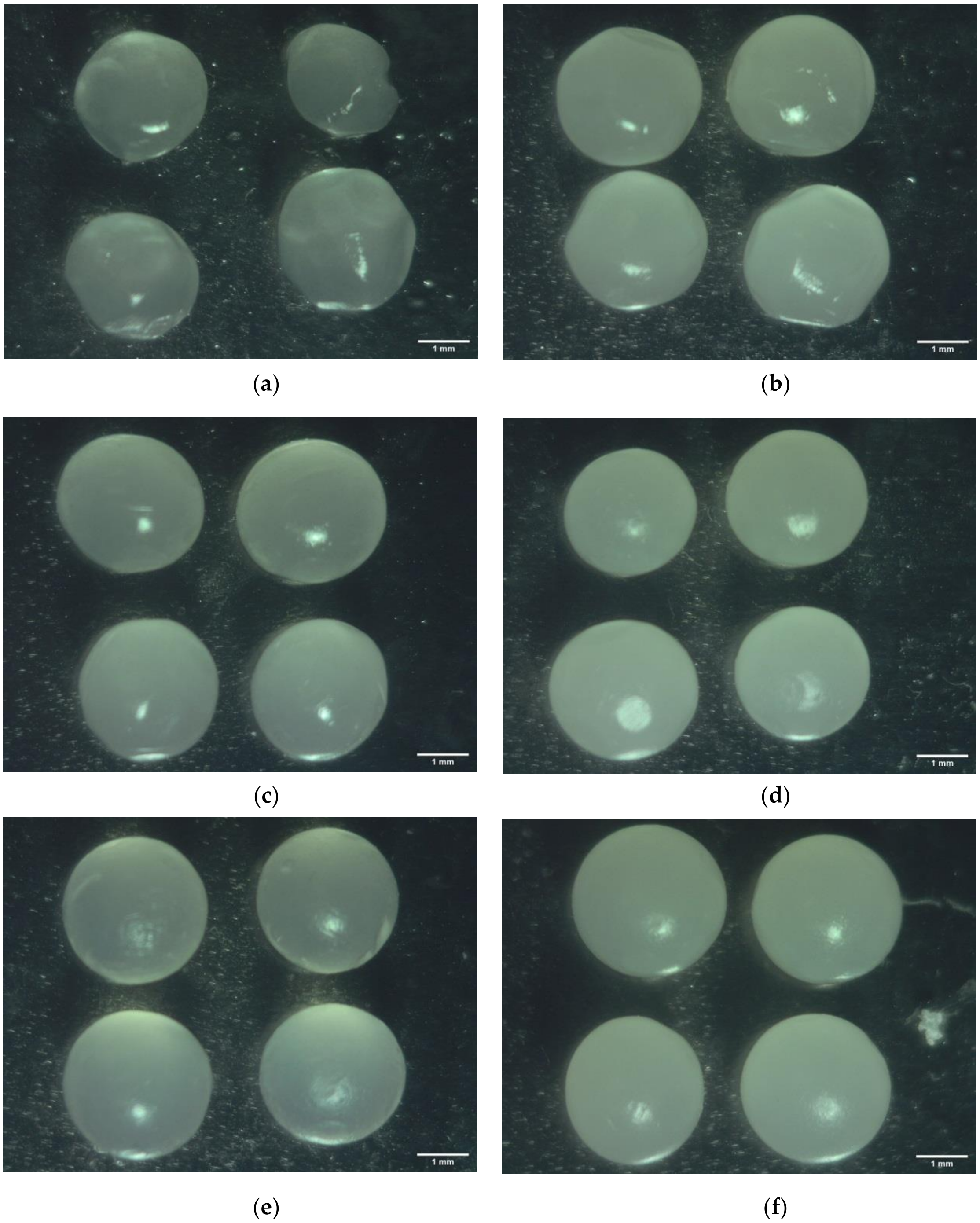

Figure S4. Microphotographs of calcium alginate-cornstarch hydrogel beads. Cryoconcentrated from cycle 2 with: (**a**) alginate at 1% (w/w) and cornstarch at 0.5% (w/w); (**b**) alginate at 1% (w/w) and cornstarch at 2% (w/w); (**c**) alginate at 2% (w/w) and cornstarch at 0.5% (w/w); (**d**) alginate at 2% (w/w) and cornstarch at 2% (w/w); (**e**) alginate at 2% (w/w) and cornstarch at 0.5% (w/w); (**f**) alginate at 2% (w/w) and cornstarch at 2% (w/w).

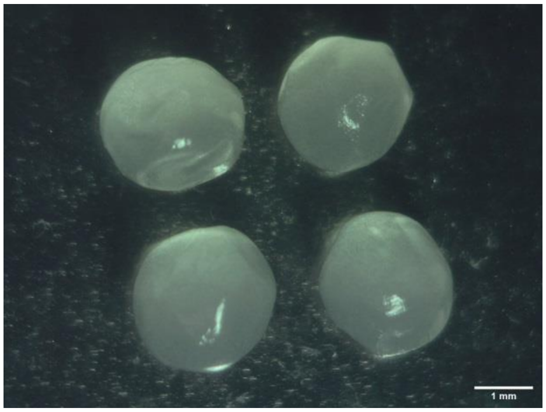

(**a**)

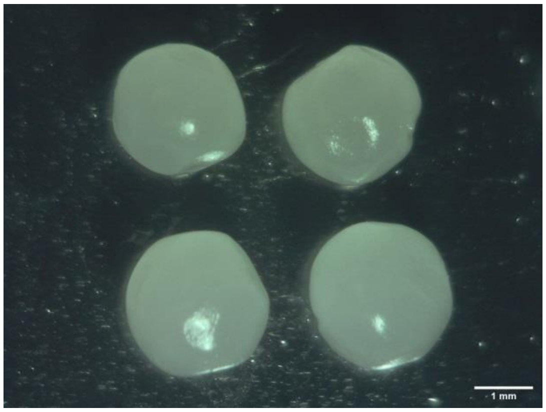

(**b**)

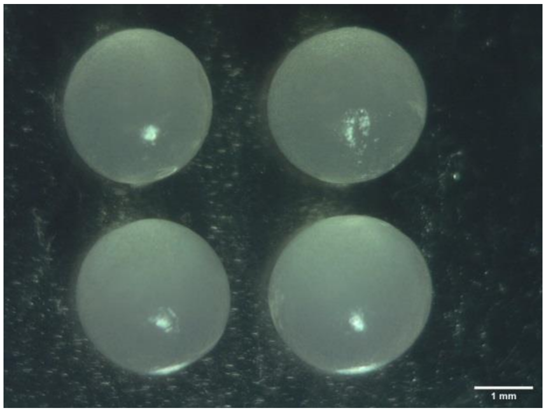

(**c**)

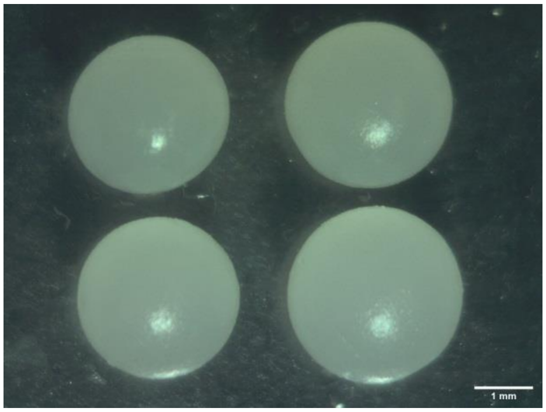

(**d**)

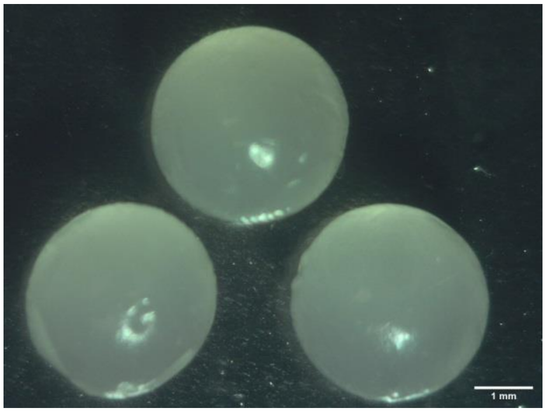

(**e**)

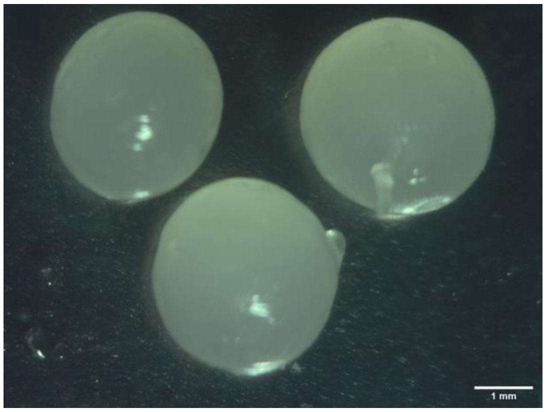

(**f**)

Figure S5. Microphotographs of calcium alginate-cornstarch hydrogel beads. Cryoconcentrated from cycle 3 with: (**a**) alginate at 1% (w/w) and cornstarch at 0.5% (w/w); (**b**) alginate at 1% (w/w) and cornstarch at 2% (w/w); (**c**) alginate at 2% (w/w) and cornstarch at 0.5% (w/w); (**d**) alginate at 2% (w/w) and cornstarch at 2% (w/w); (**e**) alginate at 2% (w/w) and cornstarch at 0.5% (w/w); (**f**) alginate at 2% (w/w) and cornstarch at 2% (w/w).

Table S1. Main results of calcium alginate (2% w/w)-cornstarch (2% w/w) hydrogel beads.

Sample	EE (%)	LC (%)	Particle size (mm)	SF	a_w	Moisture content (%)	ρ_B (g/mL)
Initial solution	63.28 ± 1.54^{a}	86.03 ± 0.96^{a}	3.09 ± 0.02^{a}	0.02 ± 0.002^{a}	0.98 ± 0.01^{a}	86.1 ± 0.15^{a}	1.04 ± 0.05^{a}
Cycle 1	90.17 ± 3.15^{c}	81.12 ± 0.84^{c}	2.82 ± 0.02^{b}	0.02 ± 0.003^{ab}	0.97 ± 0.00^{ab}	81.5 ± 0.56^{b}	1.05 ± 0.05^{ab}
Cycle 2	97.73 ± 1.38^{d}	74.52 ± 1.02^{d}	2.73 ± 0.03^{c}	0.02 ± 0.004^{ab}	0.95 ± 0.01^{c}	80.0 ± 0.10^{bc}	1.11 ± 0.03^{bc}
Cycle 3	75.09 ± 3.01^{b}	68.25 ± 0.45^{b}	2.64 ± 0.02^{d}	0.02 ± 0.002^{a}	0.92 ± 0.01^{d}	73.6 ± 0.23^{d}	1.16 ± 0.11^{abcd}

Different small letters indicate significant differences at $p < 0.05$, respectively.
